# The re-emergence of dengue virus in non-endemic countries: a case series

**DOI:** 10.1186/1756-0500-7-596

**Published:** 2014-09-03

**Authors:** Danilo Buonsenso, Giovanni Barone, Roberta Onesimo, Roberta Calzedda, Antonio Chiaretti, Piero Valentini

**Affiliations:** Pediatric Infectious Diseases Unit, Department of Pediatrics, Catholic University A. Gemelli Hospital, L.go A. Gemelli 8, 00168 Rome, Italy

**Keywords:** Dengue, Children, Travel, Imported dengue, Vaccine

## Abstract

**Background:**

Dengue has been designated a major international public health problem by the World Health Organization. It is endemic in most tropical and sub-tropical countries, which are also popular tourist destinations. Travelers are at significant risk of acquiring the disease and also contribute to its spread to non-endemic countries where the vector is present. Children represent a particular susceptible category, since they have a higher risk than adults of developing severe dengue.

**Case presentation:**

We describe 3 cases of imported dengue fever in Italy in three children (two born in the Philippines and one of Bangladeshi ethnicity) who acquired dengue fever during a recent travel to Southeast Asia, initially not-recognized because of the low index of suspicion of physicians not working in dengue endemic areas. Clinical presentations, differential diagnosis and management of these children are presented and discussed.

**Conclusions:**

Due to global urbanization and increased air travel, it is nowadays important that physicians who practice outside of traditionally dengue endemic areas are adept at the recognition of potentially fatal reemerging infectious diseases such as dengue.

**Electronic supplementary material:**

The online version of this article (doi:10.1186/1756-0500-7-596) contains supplementary material, which is available to authorized users.

## Background

Dengue has been designated a major international public health problem by the World Health Organization (WHO). It is endemic in most tropical and sub-tropical countries, which are also popular tourist destinations. Travelers are at significant risk of acquiring the disease and also contribute to its spread to non-endemic countries [1]. They may further serve as sentinels to alert the international community to epidemics in dengue-endemic regions and to the spread of dengue virus serotypes and genotypes [2]. Children represent a particular susceptible category, since they have a higher risk than adults of developing severe dengue (up to 10% cases) [3].

We describe 3 cases of imported dengue fever in Italy in three children who acquired Dengue fever during a travel to Southeast Asia in 2013. The purpose of this report is to review current literature regarding the epidemiology, clinical manifestations, diagnosis, and management of this important, still unrecognized global remerging infection, which has particular impact on the pediatric population.

## Cases presentation

### Case 1

**Figure 1:**
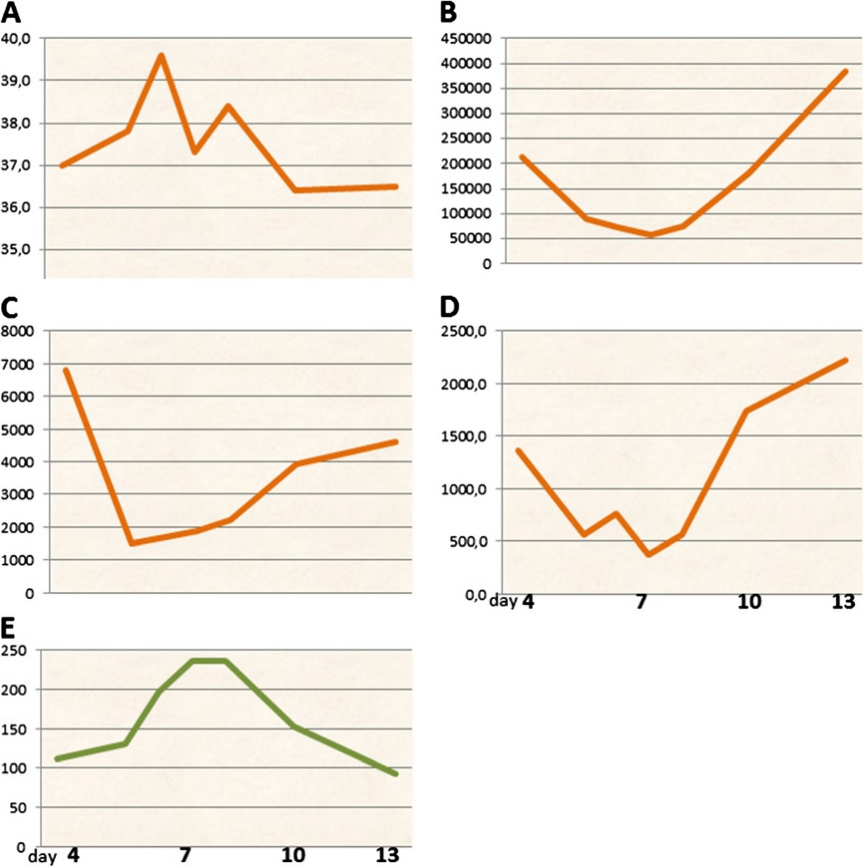
**Schematic representation of main laboratory tests performed to patient 1. A**: hematocrit; **B**: platelets; **C**: white cells; **D**: neutrophils; **E**: transaminasis (sGPT/ALT).

A previously healthy 10-year-old boy born in the Philippines presented to a pediatric emergency department with a 3-day history of fever (max temperature 39.6°C), generalized malaise, severe headache, vomiting and weight loss. His parents reported that the baby had been in the Philippines to meet his grandparents and was back in Italy since 5 days. On admission, the patient was alert and had a temperature of 39.9°C, heart rate of 110 beats per minute, respiratory rate of 20 breaths per minute, and a blood pressure of 104/60 mm Hg. Physical examination revealed a suffering teenager with a mild macula-papular rash, rigor nucalis, severe back pain and myalgia, abdominal tenderness and retro-orbital pain during eye movements. He did not have lymphadenopathy, or visceromegaly. Laboratory data included a white blood cell count of 6800/mL (with 82% neutrophils, 8% lymphocytes, and 10% monocytes), hemoglobin level of 12.8 g/dL, platelet count of 214,000/mm^3^, an aspartate aminotransferase level of 262 U/L, an alanine aminotransferase level of 196 U/L, C-reactive protein of 38 mg/dL (normal value < 0.5 mg/dl). Serum urea nitrogen and electrolytes, serum creatinine, urinalysis and coagulation studies were within reference limits. Chest X-ray was normal. Lumbar puncture was performed due to a suspected central nervous system infection, but cerebrospinal fluid indices were normal. Head computed tomography (CT) was negative. Thick and thin stained blood smears were negative for malaria. The parents reported that dengue hemorrhagic fever (DHF) was ongoing in the Philippines. At this point, a blood specimen for dengue fever serology and reverse transcription-polymerase chain reaction was submitted to the Italian National Institute of Health and the patient was transferred to the Pediatric Infectious Disease Unit of our Institution. Due to the potential exposition to a dengue fever epidemics, this diagnosis was considered as the most probable. Nevertheless, differential diagnosis at this time still included viral syndrome, meningococcemia, and Rocky Mountain spotted fever (RMSF). Serology for Epstein-Barr virus (EBV), Cytomegalovirus (CMV), coxsackie virus, echovirus, hepatitis A virus, RMSF, *Ehrlichia chaffeensis*, and *Coxiella burnetti* were performed. The fever had continued for 4 days and subsided on the second day of hospitalization. On the second day of admission, one episode of coffee ground vomiting developed, thrombocytopenia proceeded to 50000/mm^3^ at the minimum on day 6, the white blood cell count exhibited a minimum of 1,800/dL on day 5 (400 neutrophils/dL), and all these parameters began to rise on day 8 of illness. Haematocrit peaked up to 40% on day 6 and decreased to 36% on day 10. The transaminase level peaked on days 5 to 8 of his illness (both aspartate aminotransferase and alanine aminotransferase level up to 250 IU/L) (Figure 1). Urine output, blood pressure and heart rate remained always within normal ranges. Results of serology for EBV, CMV, RMSF, *Ehrlichia chaffeensis*, and *Coxiella burnetti* were all negative. Cerebrospinal fluid, blood, fecal, and urine cultures were sterile. Result of throat viral culture was negative. A recent dengue infection was confirmed by demonstration of immunoglobulin M (IgM) antibody and positive RT-PCR for dengue virus type 2 and intravenous ceftriaxone and acyclovir were discontinued. Intravenous fluid therapy was stopped when the recovery of appetite, decrease in hematocrit and increase in platelet count was observed. The child gradually improved, bradicardia developed (up to 50 bpm) and was discharged on the tenth day of hospitalization. At the 2-week follow-up visit, the patient was well appearing with resolution of thrombocytopenia, leukopenia, and hepatitis.

### Case 2

The second case was a 1-year-old Bangladeshi girl who visited her relatives in Bangladesh in October 2012. During the last days before coming in Italy she suffered from fever (39°C), myalgias and nausea and was diagnosed with dengue virus infection in her birth country. Due to an uncomplicated disease, the girl was not admitted to the hospital and the girl and her family took the flight to Italy. Four days later, due to persistence of high-grade fever, she presented to the emergency ward in our hospital and admitted to the pediatric infectious disease unit. On admission, the body temperature was 38°C, physical examination was normal. Blood cultures were negative for bacteria and malaria was ruled out by microscopic blood examination. Serology was positive for dengue virus (via enzyme-linked immunosorbent assay [ELISA]). Serology for human immunodeficiency virus (HIV) infection, acute hepatitis A, B and C, CMV and EBV were negative. Haematocrit, white-blood cell, platelet count, and liver-function tests remained always normal. The girl was able to retain oral fluids and diuresis was valid. The girl had non-complicated dengue fever, according to the new classification of the World Health Organization [4].

### Case 3

The third case was a 9-year-old Philippine girl resident in Italy, who went for holiday with her family to the Philippines. During her stay in the Philippines she developed high grade fever (up to 39.5°C), treated with paracetamol for three days with benefit, and diarrhea. On the fourth and fifth day she developed epistaxis and was brought to the emergency room in her birth country were dengue virus infection was diagnosed. The day after, despite the persistence of fever, the girl and the family took the flight to come back in Italy. Due to the persistence of fever on the sixth day, the family took the girl to the emergency room of our hospital. On admission, the girl appeared in good clinical condition, the body temperature was 37.8°C, physical examination was normal. The girl had signs of mild dehydration. Laboratory data included a white blood cell count of 8630 cells/ml (with 69.8% neutrophils, 21.3% lymphocytes and 8.6% monocytes), hemoglobin level of 12.6 g/dL, platelet count of 175.000/mm^3^, C-reactive protein of 3.54 mg/dl (normal value < 0.5 mg/dl). Serum urea nitrogen and electrolytes, serum creatinine, aminotransferases, urinalysis and coagulation studies were within reference limits. Chest X-ray was normal. The patient was then admitted to the Pediatric Infectious Disease Unit of our Institution and intravenous fluids were started. Due to a recent travel to a dengue-endemic country, a blood specimen for dengue fever serology and reverse transcription-polymerase chain reaction was submitted to the Italian National Institute of Health, which turned positive for dengue virus type 2. During hospitalization haematocrit, white-blood cell, platelet counts and liver-function tests remained always normal. Fever subsided on the 8th day of illness. Urine output, blood pressure and heart rate remained always within normal ranges for age. The girl did not present new hemorrhagic episodes and was able to tolerate oral intake since the second day of admission and was discharged 11 days after disease onset, 72 hours after fever resolution.

## Discussion

### Epidemiology

Dengue fever is an acute viral illness transmitted most commonly by *Aedes aegypti* mosquitoes, which is endemic in tropical Asia, Latin America, and the Caribbean [5]. Dengue is the most rapidly spreading mosquito-borne viral disease in the world. In the last 50 years, incidence has increased 30-fold with increasing geographic expansion to new countries and, in the present decade, from urban to rural settings. An estimated 50 million dengue infections occur annually and approximately 2.5 billion people live in dengue endemic countries [6]. Because of global urbanization with substandard living conditions, increased mobility of populations, ineffective vector control, viral and vector evolution, and climate change, dengue has become a major public health challenge in the past half century [7]. In Italy, a recent retrospective study identified dengue infection in 15 out of 91 travelers from endemic areas (16.5%) [8]. This high rate of imported dengue infection in Italy, as well as in other temperate European and non European countries, where dengue virus is not endemic, is of particular concern since increased mobility of populations, substandard living conditions, ineffective vector control, viral and vector evolution, and climate change could modify the present situation [9]. The alternative dengue vector, *Aedes albopictus*, seems to be spreading into temperate climates, potentially worsening the global dengue problem [5]. In Italy, for example, the dengue vector *Aedes albopticus* is represented in the Po river valley in the North West Italy, where imported cases have been recently described [8]. Children represent a significant proportion of the travelling public, accounting for 7% (1.9 million) of travelers living in the US [9]. Classic and severe dengue in children pose a significant burden on endemic countries [10]. A study of over 1500 ill pediatric travelers reporting to GeoSentinel Clinics in 19 countries identified dengue and typhoid fever as the most frequent causes of systemic febrile illness in children returning from tropical regions other than sub-Saharan Africa. Children have a higher risk than adults of developing severe dengue, a leading cause of morbidity and death in this age-group [3], mainly for a secondary infection in which the mortality rate is nearly 15-fold higher than in adults [2, 11].

### Clinical manifestations and management

The clinical manifestations of dengue fever are varied [5]. After an incubation period of 3 to 14 days, the individual may be asymptomatic or may present with mild undifferentiated fever to severe disease that includes hemorrhagic and hypovolemic shock [2, 5]. Nevertheless, bleeding manifestations may be present in approximately 10% of cases of uncomplicated dengue fever, therefore the WHO proposed a new classification in 2009, which reclassifies dengue into dengue with or without warning signs and severe dengue (Additional file 1: Table S1) [4].

As reported in Additional file 1: Table S2, the disease course of DHF is classically divided into three phases: febrile, plasma leakage (critical), and convalescent (recovery) phases [12].

The first phase presents with influenza-like illness with sudden onset of high-grade fever, retro-orbital pain, severe headache, myalgia, arthralgia, and rash in approximately 50% of cases [5, 13]. Because of the presenting symptoms of severe myalgias, arthralgia, and fever, dengue fever was referred to as breakbone fever during World War II in India and Burm [14]. In this phase, as showed in case 1, differential diagnosis is wide. Despite the doctor was aware that the child recently travelled from Philippines to Italy, he did not think about travel-related illnesses and suspected a meningo-encephalitis, performing a lumbar puncture and head CT scan and beginning antibiotic and antiviral therapy. Dengue fever was suspected only when the mother reported an ongoing dengue epidemics in the Philippines. This point underlines the importance of always considering dengue fever in febrile travelers from endemic areas. During this phase, once dengue fever is suspected, on the basis of medical history, physical examination and full blood count and haematocrit, clinicians should be able to determine whether the disease is dengue, which phase it is in, whether there are warning signs, the hydration and hemodynamic status of the patient, and whether the patient requires admission to the hospital. To strengthen the importance of anamnesis and clinical symptoms of children suffering from dengue fever here we report the Additional file 1: Table S3.

During the febrile phase, patients who are able to tolerate adequate volumes of oral fluids and pass urine at least once every six hours, and do not have any of the warning signs, particularly when fever subsides, may not be admitted if the family is compliant, but should be reviewed daily for disease progression (decreasing white blood cell count, and warning signs) until they are out of the critical period. Those with stable haematocrit can be sent home, but, because of concerns about family compliance, we admitted to the hospital the girl of case 2 till complete resolution of dengue fever. These patients are classified as having non complicated dengue fever, as happened to the girl in case 2. Anyway, doctors dealing with a case of non complicated dengue fever should [12]

- Encourage oral intake of oral rehydration solution (ORS), fruit juice and other fluids containing electrolytes and sugar to replace losses from fever and vomiting.
- Give paracetamol for high fever if the patient is uncomfortable, avoiding acetylsalicylic acid, ibuprofen or other non-steroidal anti-inflammatory agents (NSAIDs) as these drugs may aggravate gastritis or bleeding.
- Instruct the care-givers that the patient should be brought to hospital immediately if any of the following occur: no clinical improvement, deterioration around the time of defervescence, severe abdominal pain, persistent vomiting, cold and clammy extremities, lethargy or irritability/restlessness, bleeding (e.g. black stools or coffee-ground vomiting), not passing urine for more than 4–6 hours.
- As showed in case one, severe pain is usually one of the main symptoms of dengue fever in children. In case paracetamol would not be sufficient for pain control, some authors have demonstrated the safety and efficacy of intravenous fentanyl administered by “patient controlled anesthesia” plus background infusion in children [15].

Around the time of defervescence, when the temperature drops to 37.5–38°C or less and remains below this level, usually on days 3–7 of illness, an increase in capillary permeability in parallel with increasing haematocrit levels may occur [7, 8]. This marks the beginning of the critical phase. The period of clinically significant plasma leakage usually lasts 24–48 hours [4]. The child in case 1 had advanced to the plasma leakage phase on day 5, when he developed hemoconcentration, thrombocytopenia and leucopenia and bleeding tendencies (coffee ground vomiting). This is a critical teaching point, since clinicians must be aware that the massive capillary leak syndrome is usually noted soon after the patient is not anymore febrile (days 3 to 6), as happened in case 1, so the abrupt resolution of fever (and the apparent improvement of clinical conditions) is an indication for closer monitoring, not for discharge. A drop in platelets, accompanied or followed by a rise in hematocrit, is a worrisome finding and may precede the onset of shock. Abdominal pain, persistent vomiting, hypothermia, and restlessness are signs of impending shock. Hematocrit levels should be measured from day 3 until the patient has been afebrile for 1 to 2 days, at which time the risk of progression to shock is low. Critical points for the management of this phase are [4]:

- Give paracetamol for high fever if the patient is uncomfortable. The interval of paracetamol dosing should not be less than six hours. Do not give acetylsalicylic acid (aspirin), ibuprofen or other non-steroidal anti-inflammatory agents (NSAIDs) as these drugs may aggravate gastritis or bleeding.
- Clinical status and hematocrit need to be continuously monitored, in order to continuously review intravenous fluid therapy during the critical phase and once hemodynamic status has stabilized, to promptly recognize and treat the onset of shock, or to recognize the potential complication of fluid overload.
- Obtain a reference haematocrit before fluid therapy. Give only isotonic solutions such as 0.9% saline, Ringer’s lactate, or Hartmann’s solution. Start with 5–7 ml/ kg/hour for 1–2 hours, then reduce to 3–5 ml/kg/hour for 2–4 hours, and then reduce to 2–3 ml/kg/hour or less according to the clinical response.
- Reassess the clinical status and repeat the haematocrit. If the haematocrit remains the same or rises only minimally, continue with the same rate (2–3 ml/kg/hour) for another 2–4 hours. If the vital signs are worsening and haematocrit is rising rapidly, increase the rate to 5–10 ml/kg/hour for 1–2 hours. Reassess the clinical status, repeat the haematocrit and review fluid infusion rates accordingly.
- Give the minimum intravenous fluid volume required to maintain good perfusion and urine output of about 0.5 ml/kg/hr. Intravenous fluids are usually needed for only 24–48 hours. Reduce intravenous fluids gradually when the rate of plasma leakage decreases towards the end of the critical phase. This is indicated by urine output and/or oral fluid intake that is/are adequate, or haematocrit decreasing below the baseline value in a stable patient.
- Patients with warning signs should be monitored by health care providers until the period of risk is over. A detailed fluid balance should be maintained. Parameters that should be monitored include vital signs and peripheral perfusion (1–4 hourly until the patient is out of the critical phase), urine output (4–6 hourly), haematocrit (before and after fluid replacement, then 6–12 hourly), blood glucose, and other organ functions (such as renal profile, liver profile, coagulation profile, as indicated).

Also the girl in case 3 presented dengue fever with warning signs, since she presented mucosal bleeding for two days; nevertheless, differently from the child in case 1, girl in case 3 improved earlier and did not present a drop in platelet count and a rise in hematocrit after fever resolution. Despite adequate management, patients can progress to severe dengue fever (critical phase). All patients with severe dengue should be admitted to a hospital with access to intensive care facilities and blood transfusion. The recovery phase begins if the patient survives the 24–48 hour critical phase. General well-being improves, appetite returns, gastrointestinal symptoms abate, hemodynamic status stabilizes and diuresis ensues [4]. As shown in case 1, intravenous fluids were stopped when the child recovered appetite; this is a critical teaching point for the management of patients in the “recovery phase”, since a potential complication of this phase of dengue fever is hypervolaemia if intravenous fluid therapy has been excessive and/or has extended into this period. Despite fluid therapy is the mainstay of the management of dengue fever, a detailed description of doses and type of fluids to use in every moment of each phase or complication of dengue fever is not the purpose of this work, but readers can refer to WHO 2009 guidelines for these details [4]. What we think is particularly important for every pediatrician is the knowledge of some key clinical points, which are summarized in the following “good and bad clinical practice” table for the management of dengue fever, adapted from WHO 2009 guidelines (Additional file 1: Table S4).

### Diagnosis

The diagnosis of dengue fever is confirmed by serologic tests defined by a 4-fold increase in acute - and convalescent – phase anti-dengue IgG titers or detection of IgM to dengue virus. Culture of the virus is technically difficult. Amplification of dengue RNA by reverse transcription-polymerase chain reaction is available in research settings for confirmation and surveillance [16]. Because a definitive laboratory diagnosis of dengue fever takes more than a week, a clinical diagnosis must be made after ruling out other infectious diseases and empiric therapy started, as happened in case 1. Because the incubation period of dengue is between 3 and 14 days, fever beginning more than 2 weeks in a returned traveler after leaving an endemic region rules out the diagnosis of dengue infection (14). Infection with an individual virus provides permanent immunity to that serotype, but no immunity is conferred to the other serotypes [2, 5, 14].

### Prevention - Vaccine

There is currently no licensed dengue vaccine, and measures such as vector control are proving inadequate in reducing the incidence of the disease [17]. Therefore, with only supportive treatment of dengue available, protection against dengue is limited to avoidance of mosquito bites with the use of insect repellents, protective clothing and insecticides [2, 18]. While this could be performed in endemic areas particularly during epidemics, it could be difficult to routinely perform vector prevention measures in non endemic areas. The case of the epidemic outbreak of Chikungunya virus (CHIKV) infection in a narrow area of the Romagna region in northern Italy is a key lesson on this regard [19]. The initial source transmission was identified in an Indian immigrant resident in Romagna who returned from his home country in late June 2007 during the viremic asymptomatic stage of the infection. The extremely large population of *A.albopictus* present in this region precipitated the local transmission. A total of 248 cases were identified over a geographical area that was enlarged to most of the Romagna region (including a small 5 cases cluster in the regional capital city of Bologna located some 70 kilometers away from the origin of the epidemic). The epidemic of CHIKV infection that occurred in northern Italy constitutes a new model for the diffusion of a tropical disease outside the conventional locations. The outbreak mainly resulted from the “dangerous mixture” of the large population of a highly competent vector, the tiger mosquito, and the possibility that an individual returned from an area of normal diffusion of CHIKV during the asymptomatic viremic stage of infection. Considering the difficulties in controlling the spread of *A. albopictus* and the large population travelling to and from the areas of normal diffusion of vector-borne tropical diseases we think that the 2007 epidemic may be only the first of a possible series of these outbreaks [19]. What happened with CHIKV could happen with dengue virus too, since the vector of Dengue virus is diffused in Italy too and the high rate of international travels from dengue endemic countries could easily introduce the virus in non endemic areas. It is particularly worrisome that 2 out of the 3 children we evaluated were already diagnosed with dengue in their birth country and decided to take the flight and fly to Italy during the febrile, viremic stage of the disease, with the potential of local transmission in case of presence of large population of dengue vector in the destination area, such as Italy. This highlights a new possibility for dengue control: it could be a measure of control introducing an airport gate at least during epidemic seasons. Probably, feverish patients or at least those with a known diagnosis of dengue disease should not be allowed to take plane, at least those directed in area with the potential of local transmission due to the presence of vector, such as Italy. In fact, models describing the temporal dynamics of the *Aedes albopictus* coupled to an epidemic transmission model describing the spread of the epidemic in both humans and mosquitoes have widely supported the hypothesis that outbreaks of CHIKV and similar viruses in those temperate climate countries characterized by high density of *Aedes albopictus* are probable after the importation of an index case from abroad [20]. Due to all these difficulties in epidemiological control of dengue infection, an effective and cost-effective vaccine against dengue would therefore be a major advance in controlling the disease [21]. Given the high incidence of the disease in travelers, a vaccine for them may also be indicated, provided that it is safe, convenient to administer and affordable [22]. The vaccine candidate furthest in development is a chimeric vaccine by Sanofi Pasteur. With the lead candidate vaccine showing encouraging results in late-stage clinical trials, the outlook for introduction of a vaccine against all four dengue serotypes into national immunization programmes of endemic countries is promising [2, 23].

## Conclusions

Global urbanization and increased air travel necessitates the ability of physicians who practice outside of traditionally dengue endemic areas to become adept at the recognition of potentially fatal reemerging infectious diseases such as dengue. Dengue infection should be included in the differential diagnosis of patients with febrile illness with a history of recent travel to endemic regions. Furthermore, travelers contribute to the geographic spread of dengue and its introduction to previously uninfected areas. The rising numbers of dengue cases reported worldwide, the resurgence of *A. aegypti* in the Western Hemisphere, such as Italy, and the identification of locally acquired dengue infections in non-endemic regions, emphasize the need for surveillance of travelers returning from endemic areas. Early detection and management of severe disease is critical to prevent morbidity and mortality [24]. Due to the global diffusion of dengue virus, dengue cannot anymore be considered a disease localized only in some endemic countries, but it has to be considered a global disease that every physician could deal with during his career. Therefore, on the basis of evaluations of the history, physical examination and/or full blood count and haematocrit, all clinicians despite their specialty should be able to determine whether the disease is dengue, which phase it is in (febrile, critical or recovery), whether there are warning signs, the hydration and hemodynamic status of the patient, and whether the patient requires admission to the hospital.

### Consent

Written informed consent was obtained from the patients’ parents for publication of this Case Report and any accompanying images. A copy of the written consent is available for review by the Editor-in-Chief of this journal.

## Electronic supplementary material

Additional file 1: Table S1: WHO dengue classification. Adapted (11). AST: aspartate transaminase; ALT: alanine transaminase; CNS: central nervous system. **Table S2.** The three classic phases of dengue fever. Adapted from WHO (11). **Table S3.** Clinical-laboratoristic approach to a child with suspected dengue fever. Adapted (11). ECG: electrocardiogram. **Table S4.** Good and bad practice for a clinician dealing with a case of dengue fever. (DOCX 19 KB)
