# Supplementary material for: The re-emergence of dengue virus in non-endemic countries: a case series
Source: BMC Res Notes. 2014 Sep 3;7:596. doi: 10.1186/1756-0500-7-596 (PMC4174650; doi:10.1186/1756-0500-7-596)
Supplement: Supplementary file 1 — Additional file 1: Table S1: WHO dengue classification. Adapted (11). AST: aspartate transaminase; ALT: alanine transaminase; CNS: central nervous system. Table S2. The three classic phases of dengue fever. Adapted from WHO (11). Table S3. Clinical-laboratoristic approach to a child with suspected dengue fever. Adapted (11). ECG: electrocardiogram. Table S4. Good and bad practice for a clinician dealing with a case of dengue fever. (DOCX 19 KB) [file 13104_2013_3169_MOESM1_ESM.docx]

| **Dengue fever** | **Dengue fever with warning signs** | **Sever dengue fever** |
| --- | --- | --- |
| Probable dengue live in /travel to dengue endemic area.  Fever and 2 of the following criteria:  • Nausea, vomiting  • Rash  • Aches and pains  • Tourniquet test positive  • Leukopenia  • Any warning sign  Laboratory-confirmed dengue  (important when no sign of plasma leakage) | • Abdominal pain or tenderness  • Persistent vomiting  • Clinical fluid accumulation  • Mucosal bleed  • Lethargy, restlessness  • Liver enlargement >2 cm  • Laboratory: increase in HCT  concurrent with rapid decrease  in platelet count | Severe plasma leakage  leading to:  • Shock  • Fluid accumulation with respiratory distress  Severe bleeding as evaluated by clinician  Severe organ involvement  • Liver: AST or ALT >=1000  • CNS: Impaired consciousness  • Heart and other organs |

**Table 1.** WHO dengue classification. Adapted (11). AST: aspartate transaminase; ALT: alanine transaminase; CNS: central nervous system

| **Febrile phase** | Dehydration; high fever may cause neurological disturbances and febrile seizures in young children |
| --- | --- |
| **Critical phase** | Shock from plasma leakage; severe haemorrhage; organ impairment |
| **Recovery phase** | Hypervolaemia (only if intravenous fluid therapy has been excessive and/or has extended into this period) |

**Table 2.** The three classic phases of dengue fever. Adapted from WHO (11)

| **History** | – date of onset of fever/illness;  – quantity of oral intake;  – assessment for warning signs;  – diarrhoea;  – change in mental state/seizure/dizziness;  – urine output (frequency, volume and time of last voiding);  – other important relevant histories, such as family or neighbourhood dengue,  travel to dengue endemic areas, co-existing conditions (e.g. infancy, pregnancy, obesity, diabetes mellitus, hypertension), jungle trekking and swimming in waterfall (consider leptospirosis, typhus, malaria), recent  unprotected sex or drug abuse (consider acute HIV seroconversion illness). | |
| --- | --- | --- |
| **Physical examination** | – assessment of mental state;  – assessment of hydration status;  – assessment of haemodynamic status;  –checking for tachypnoea/acidotic breathing/pleural effusion;  –checking for abdominal tenderness/hepatomegaly/ascites;  –examination for rash and bleeding manifestations;  – tourniquet test | |
| **Investigations** | Full blood count  Haematocrit test  White blood cell count  Platelet count  Liver function, glucose, serum electrolytes, urea and creatinine, bicarbonate, lactate, cardiac enzymes, ECG and urine specific gravity.  Disease notification | should be done at the first visit  in the early febrilephase establishes the patient’s own baseline haematocrit  A decreasing white blood cell count makes dengue very likely.  A rapid decrease in platelet count in parallel with a rising haematocrit compared to the baseline is suggestive of progress to the plasma leakage/critical phase of the disease.  Evaluate organ disfunction  Laboratory confirmation is not necessary before notification, but should be obtained. |

**Table 3.** Clinical-laboratoristic approach to a child with suspected dengue fever. Adapted (11). ECG: electrocardiogram.

| **Good practice** | **Bad practice** |
| --- | --- |
| Assessment and follow-up of patients with non-severe dengue and instruction of warning signs to watch out for | Sending patients with non-severe dengue home with no follow-up and inadequate instructions |
| Administration of paracetamol for high fever if the patient is uncomfortable | Administration of acetylsalicylic acid (aspirin) or ibuprofen |
| Obtaining a haematocrit level before and after fluid boluses | Not knowing when haematocrit levels are taken  with respect to fluid therapy |
| Clinical assessment of the haemodynamic status before and after each fluid bolus | No clinical assessment of patient with respect  to fluid therapy |
| Interpretation of haematocrit levels in the context of fluid administered and haemodynamic assessment | Interpretation of haematocrit levels independent  of clinical status |
| Administration of intravenous fluids for repeated vomiting or a high or rapidly rising haematocrit | Administration of intravenous fluids to any patient  with non-severe dengue |
| Use of isotonic intravenous fluids for severe dengue | Use of hypotonic intravenous fluids for severe dengue |
| Giving intravenous fluid volume just sufficient to maintain effective circulation during the period of plasma leakage for severe dengue | Excessive or prolonged intravenous fluid  administration for severe dengue |
| Avoiding intramuscular injections in dengue patients | Giving intramuscular injections to dengue patients |
| Intravenous fluid rate and frequency of monitoring and haematocrit measurement adjusted according to the patient’s condition | Fixed intravenous fluid rate and unchanged frequency of monitoring and haematocrit measurement during entire hospitalization for severe dengue |
| Close monitoring of blood glucose | Not monitoring blood glucose, unaware of the hyperglycaemic effect on osmotic diuresis and confounding hypovolaemia |
| Discontinuation or reducing fluid therapy once haemodynamic status stabilizes | Continuation and no review of intravenous  fluid therapy once haemodynamic status stabilizes |
| Careful evaluation of clinical warning signs | No evaluation of new clinical warning signs |
| Careful evaluation of laboratory warning signs | No evaluation of laboratory warning signs |

**Table 4.** Good and bad practice for a clinician dealing with a case of dengue fever.
